# Supplementary material for: Senolytic Flavonoids Enhance Type-I and Type-II Cell Death in Human Radioresistant Colon Cancer Cells through AMPK/MAPK Pathway
Source: Cancers (Basel). 2023 May 8;15(9):2660. doi: 10.3390/cancers15092660 (PMC10177236; doi:10.3390/cancers15092660)
Supplement: Supplementary file 1 [file cancers-15-02660-s001.zip › cancers-2359865-supplementary.pdf]

Senolytic Flavonoids Enhance Type-I and Type-II Cell Death in Human Radioresistant Colon Cancer Cells through AMPK/MAPK Pathway

Maria Russo *, Stefania Moccia †, Diomira Luongo † and Gian Luigi Russo

Institute of Food Sciences, National Research Council, 83100 Avellino, Italy; stefania.moccia@isa.cnr.it (S.M.); diomira.luongo@isa.cnr.it (D.L.); glrusso@isa.cnr.it (G.L.R.)

* Correspondence: maria.russo@isa.cnr.it; Tel.: +39-0825299331

† These authors contributed equally to this work.

SUPPLEMENTARY MATERIALS

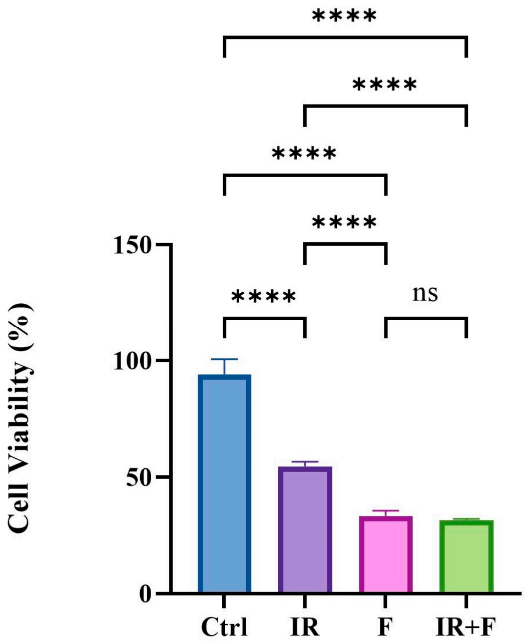

Figure S1. HT29 cells were pre-irradiated (10 Gy) and treated for 96 h with 40 µM F or IR plus F. Cell viability was performed with Cy-Quant fluorescent dye, as described [37]. Symbols indicate significance: ****p<0.0001; n.s. not significant. ANOVA Test for multiple comparisons.

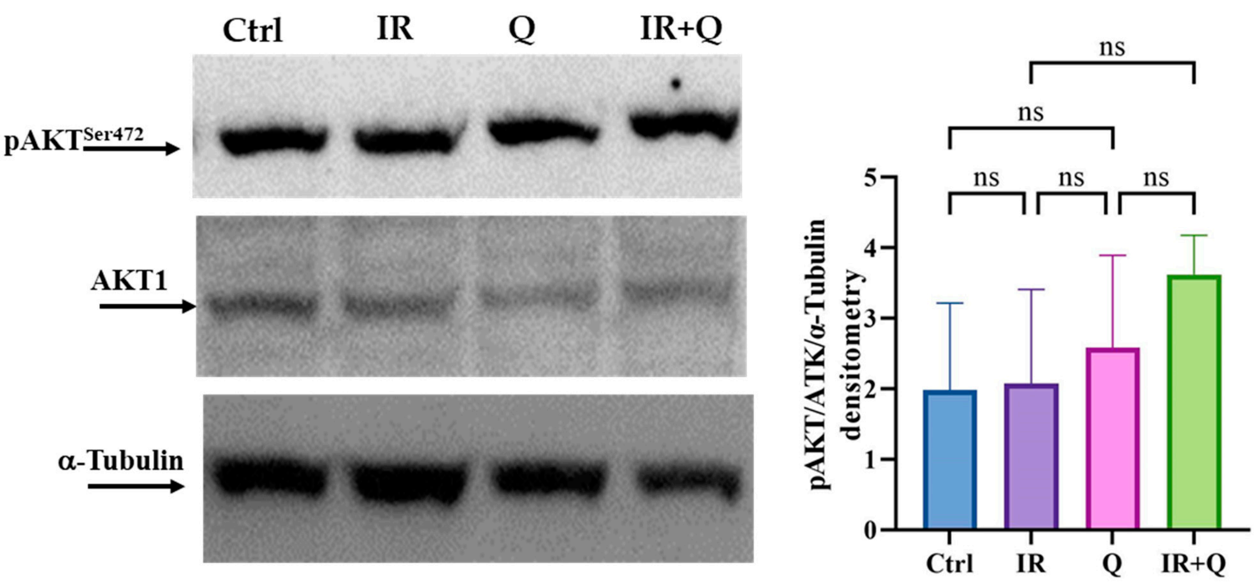

Figure S2. HT500 cells were treated with 40 µM Q or pre-irradiated (10 Gy; IR) before treatment with Q (IR plus Q) for 60 min. Cellular lysates for the immunoblots were prepared as described in the Methods section. Band intensities were quantified measuring optical density on Gel Doc 2000 and analyzed by Multi-Analyst Software; n.s. not significant. ANOVA Test for multiple comparisons.
