# Supplementary material for: Senolytic Flavonoids Enhance Type-I and Type-II Cell Death in Human Radioresistant Colon Cancer Cells through AMPK/MAPK Pathway
Source: Cancers (Basel). 2023 May 8;15(9):2660. doi: 10.3390/cancers15092660 (PMC10177236; doi:10.3390/cancers15092660)
Supplement: Supplementary file 1 [file cancers-15-02660-s001.zip › MATERIALS uncropped immunoblottings.pdf]

SENOLYTIC FLAVONOIDS ENHANCE TYPE-I AND TYPE-II CELL DEATH IN HUMAN RADIORESISTANT COLON CANCER CELLS THROUGH AMPK/MAPK PATHWAY

Maria Russo*, Stefania Moccia†, Diomira Luongo† and Gian Luigi Russo

Institute of Food Sciences, National Research Council, 83100 Avellino, Italy; stefania.moccia@isa.cnr.it (S.M.); diomira.luongo@isa.cnr.it (D.L.); glrusso@isa.cnr.it (G.L.R.)

* Correspondence: maria.russo@isa.cnr.it; Tel.: +39-0825299331

† These authors contributed equally to this work.

SUPPLEMENTARY MATERIALS: uncropped immunoblottings

Figure 2

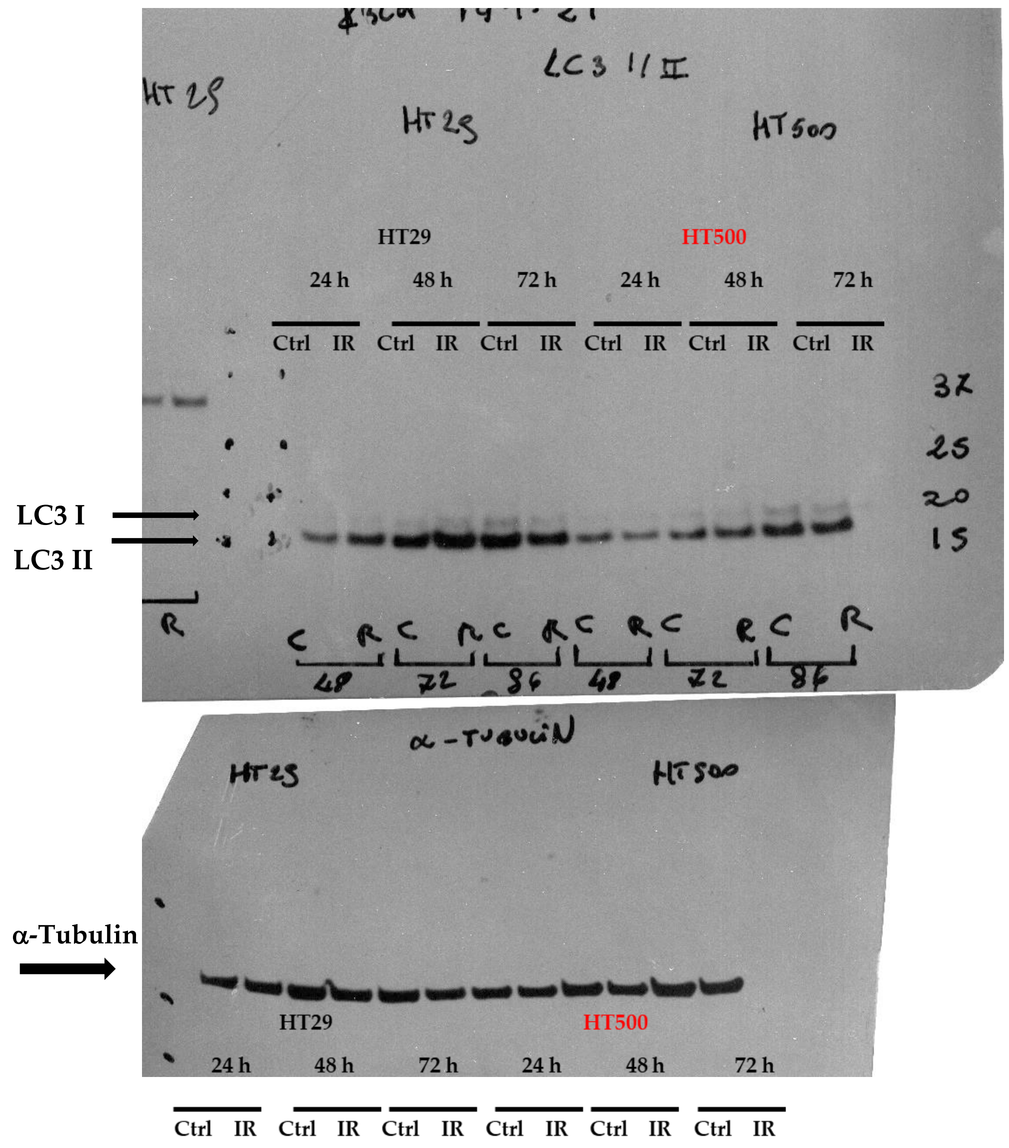
LC3 I
LC3 II
HT29
HT500
24 h 48 h 72 h 24 h 48 h 72 h
Ctrl IR Ctrl IR Ctrl IR Ctrl IR Ctrl IR Ctrl IR
α-Tubulin
HT29
HT500
24 h 48 h 72 h 24 h 48 h 72 h
Ctrl IR Ctrl IR Ctrl IR Ctrl IR Ctrl IR Ctrl IR

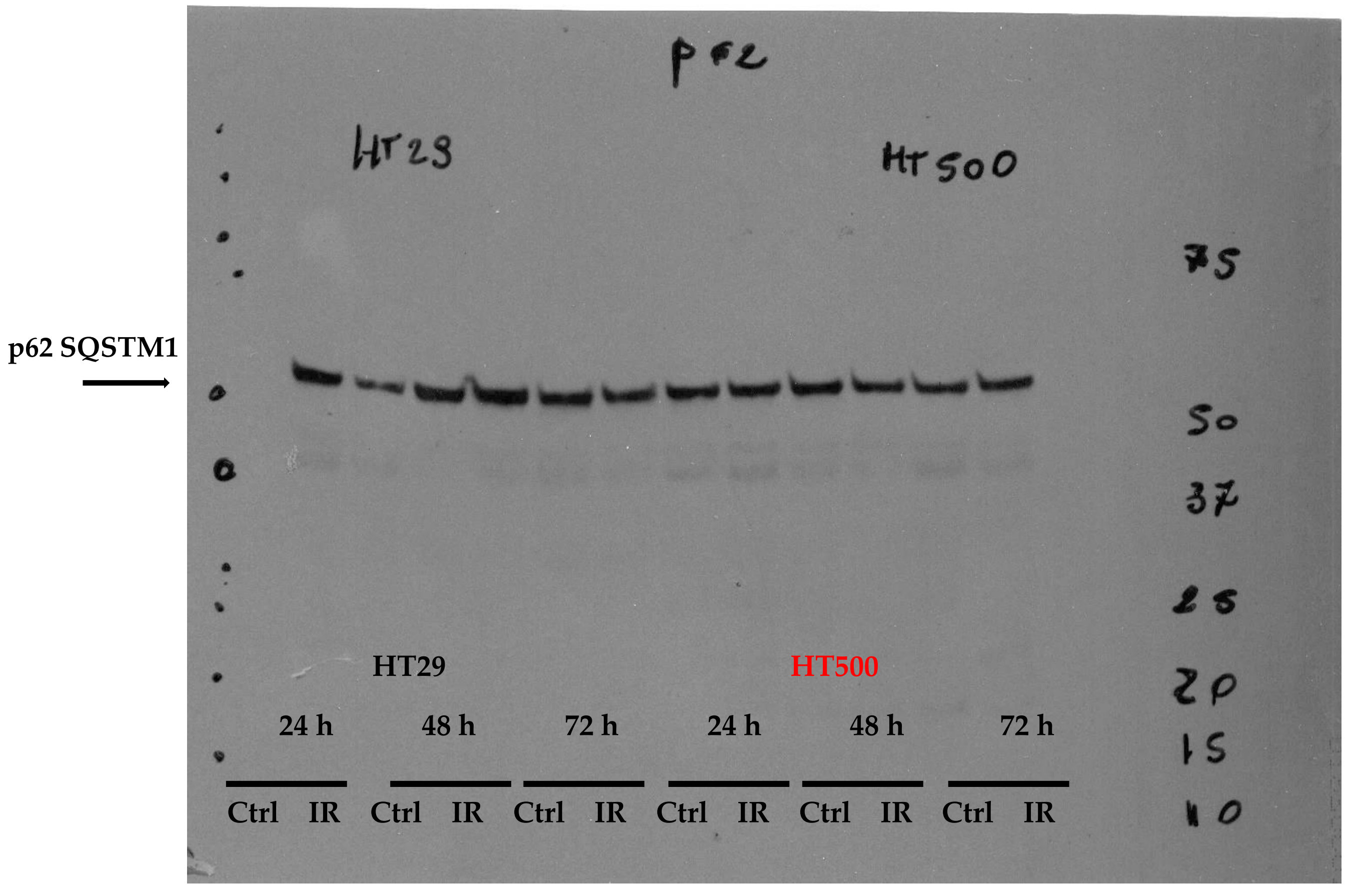
p62 SQSTM1
HT29
HT500
24 h 48 h 72 h 24 h 48 h 72 h
Ctrl IR Ctrl IR Ctrl IR Ctrl IR Ctrl IR Ctrl IR

Figure 5 a

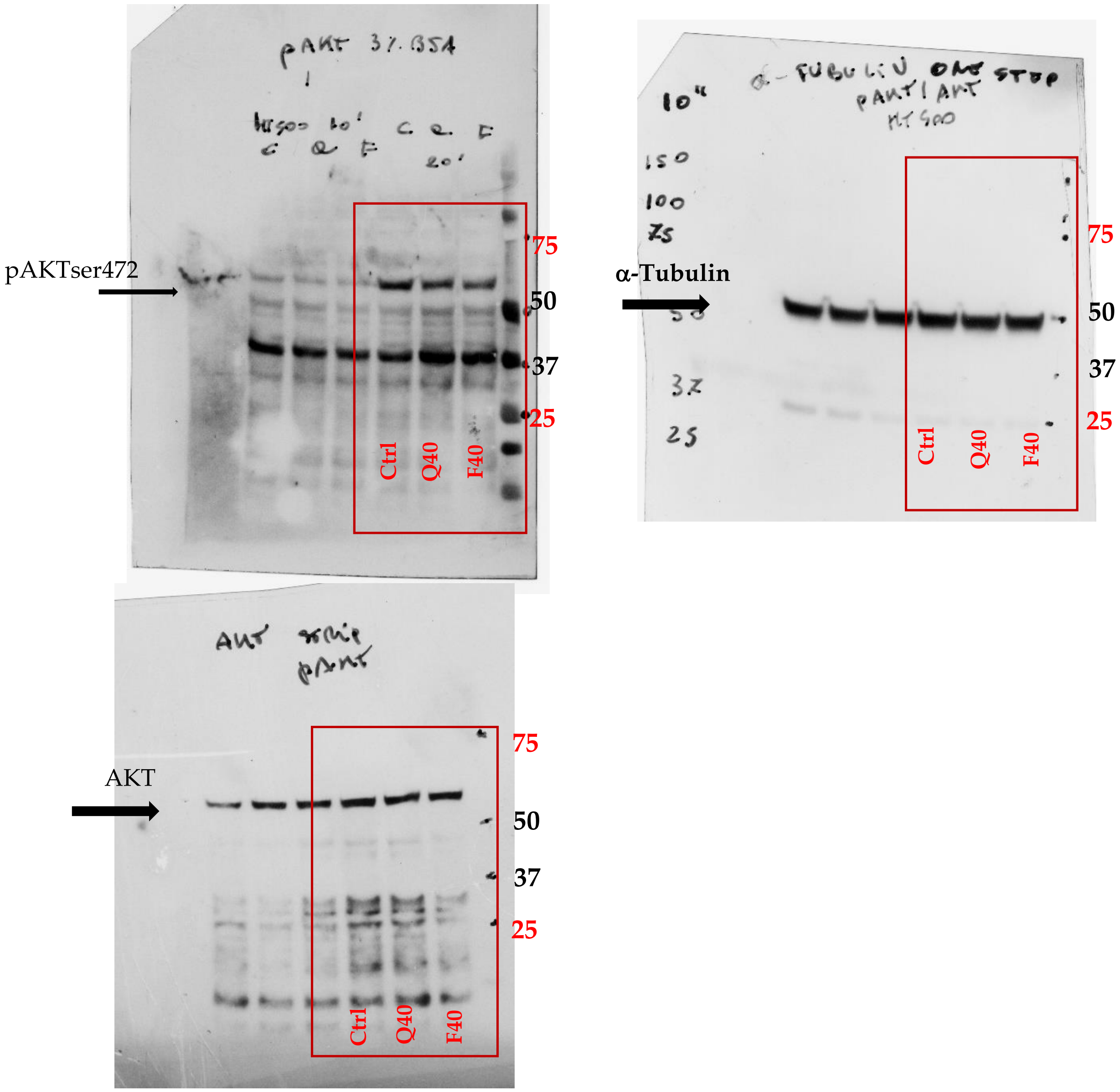

Figure 5 b

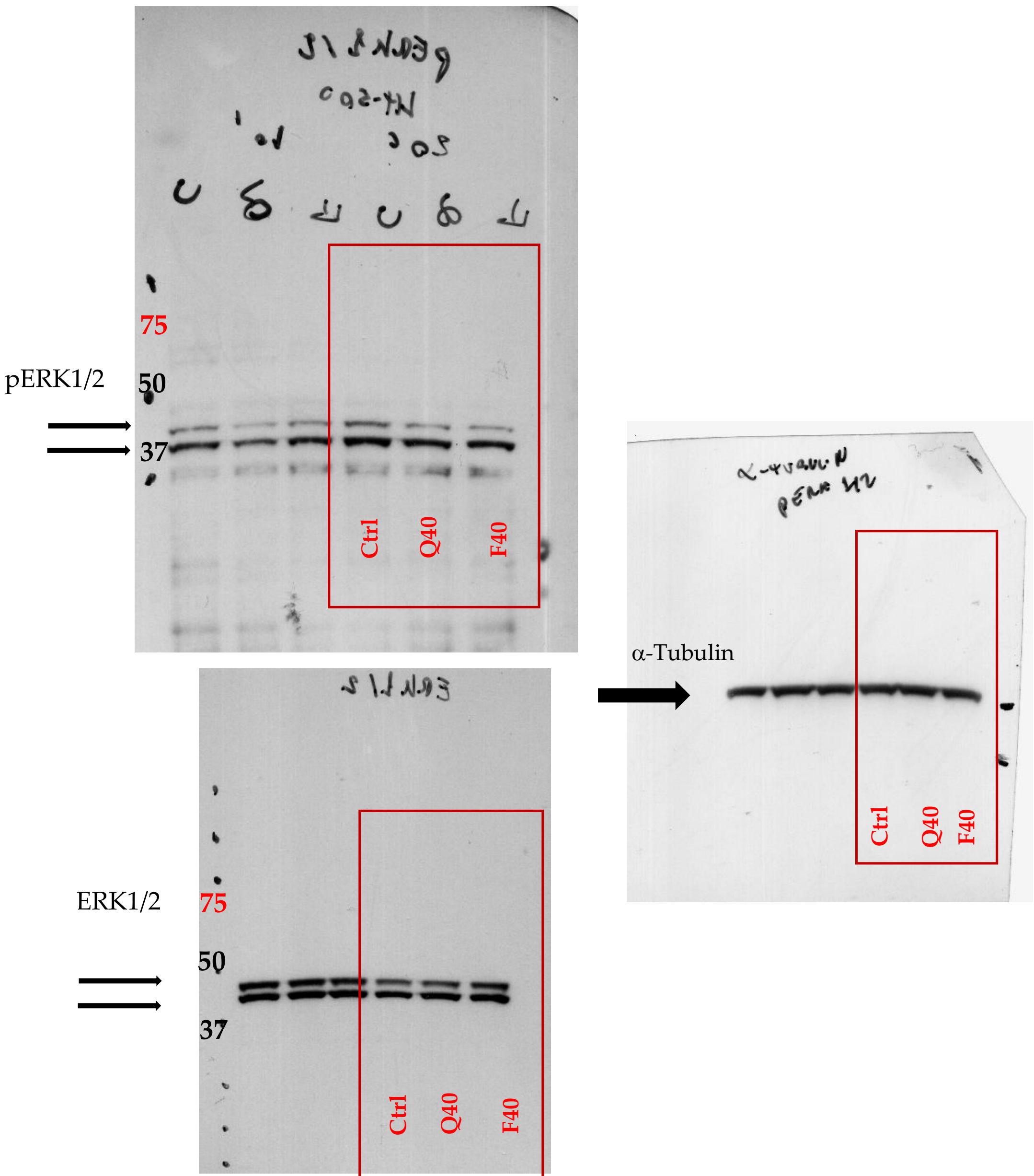

Figure 5 e

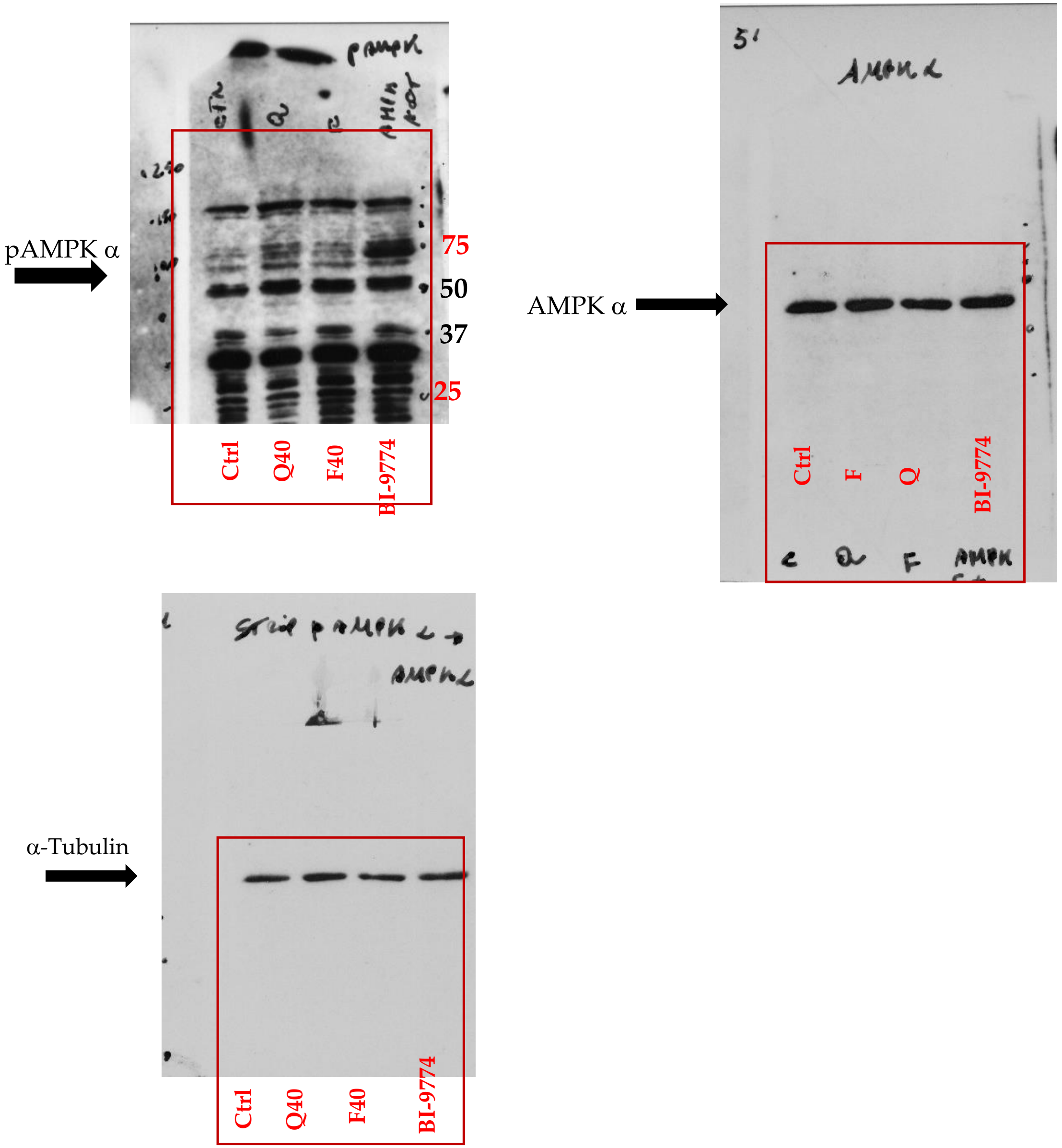

Figure 5 f

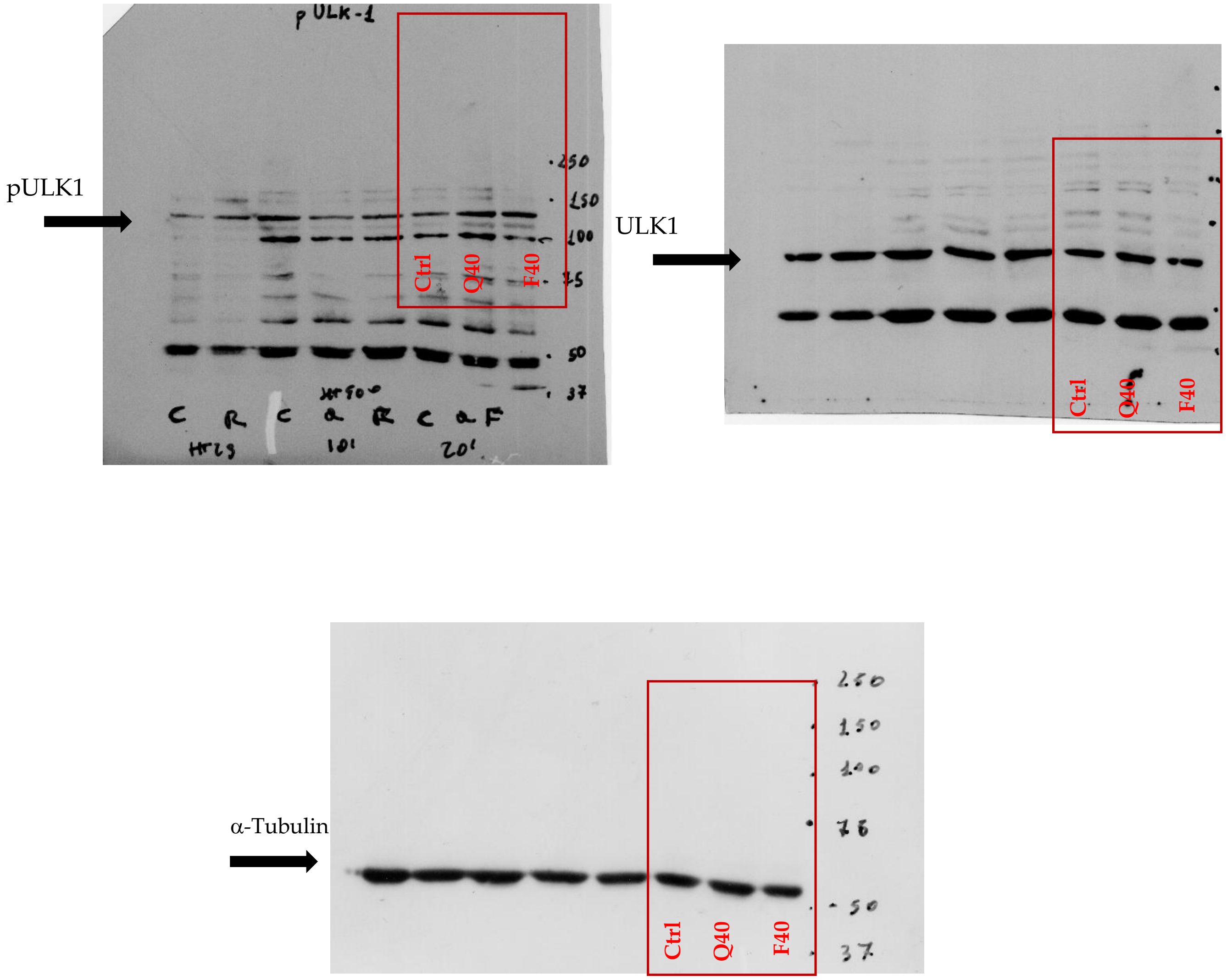

Figure 6 a

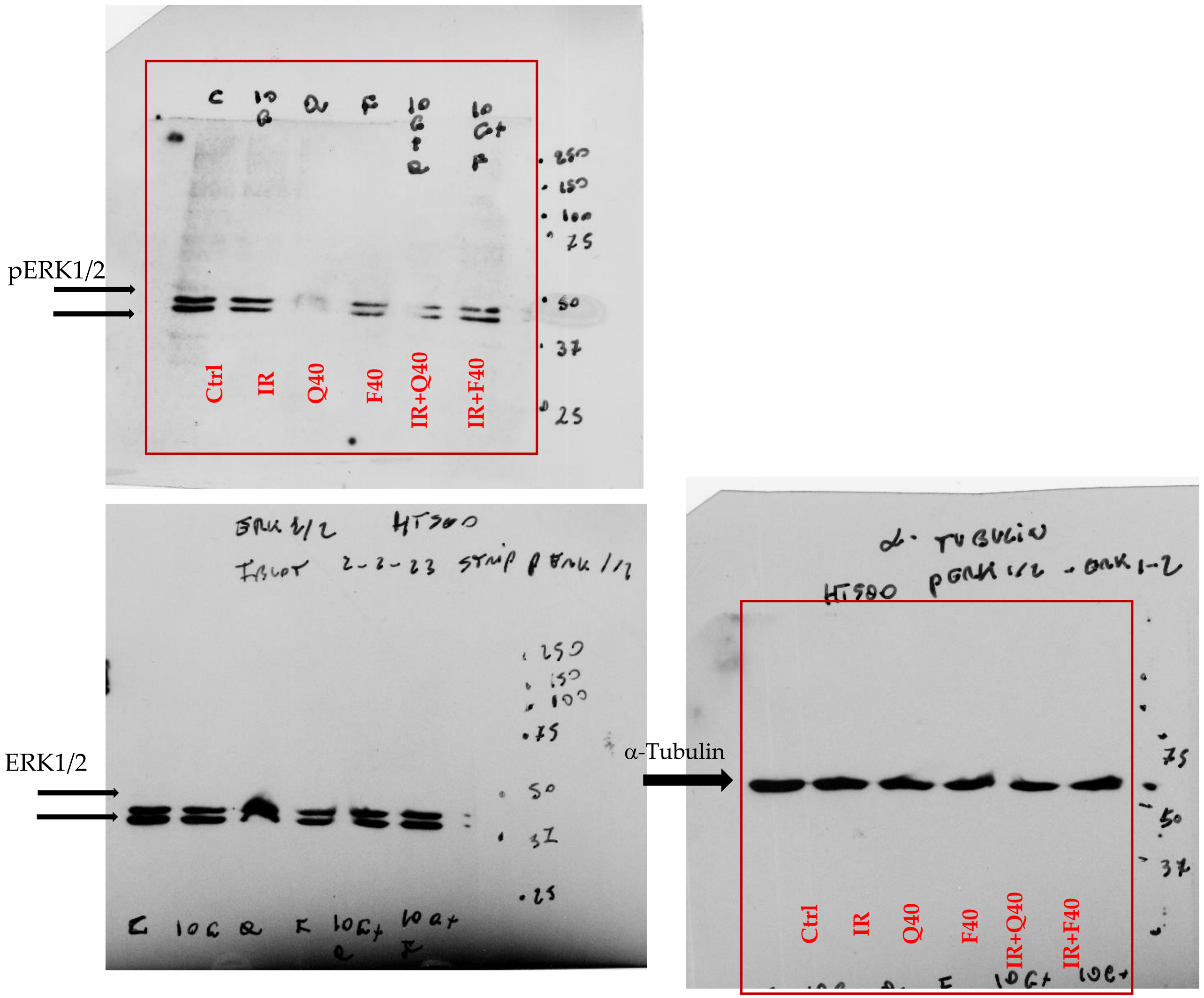

Figure 6 c

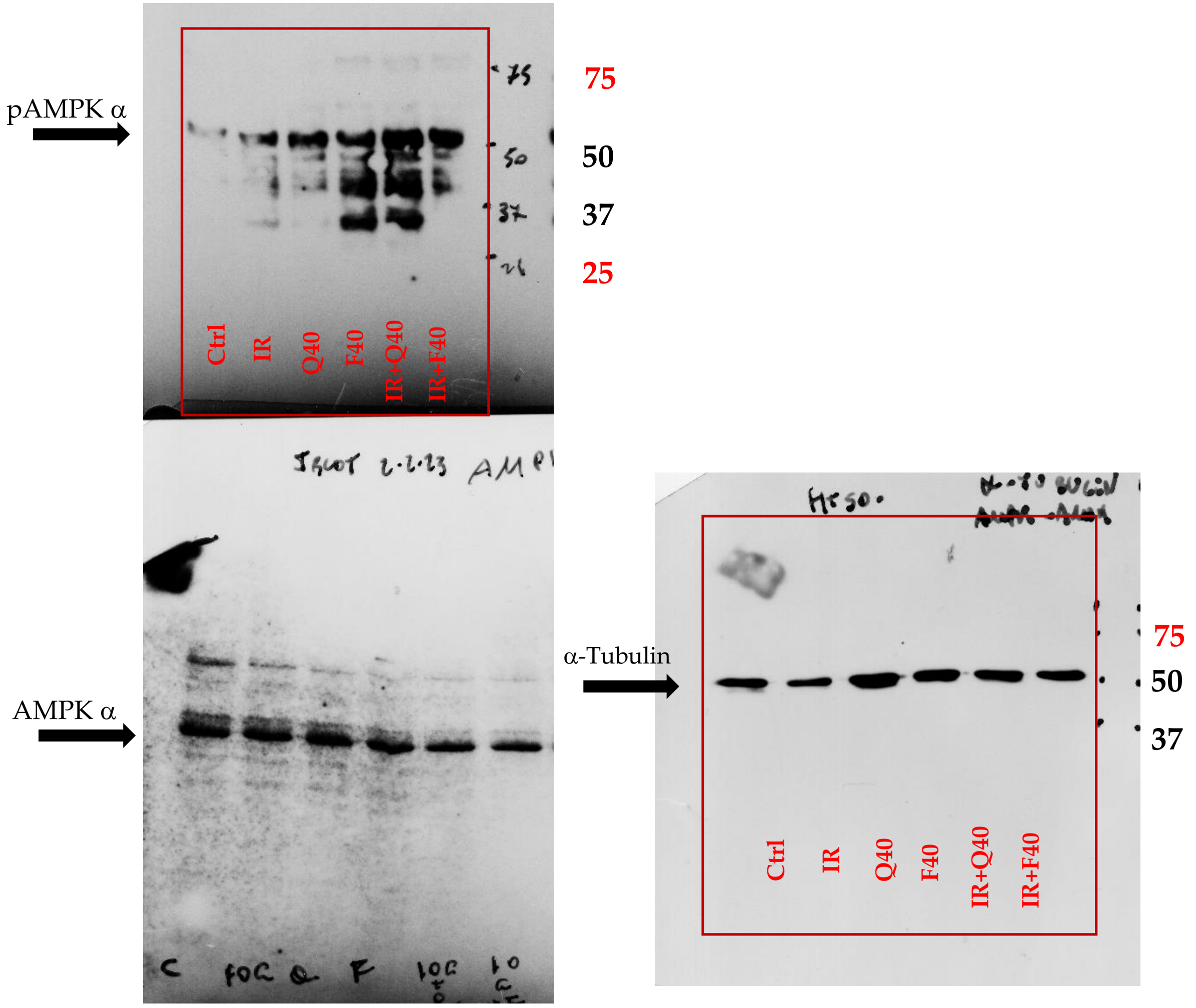

Figure 8 a

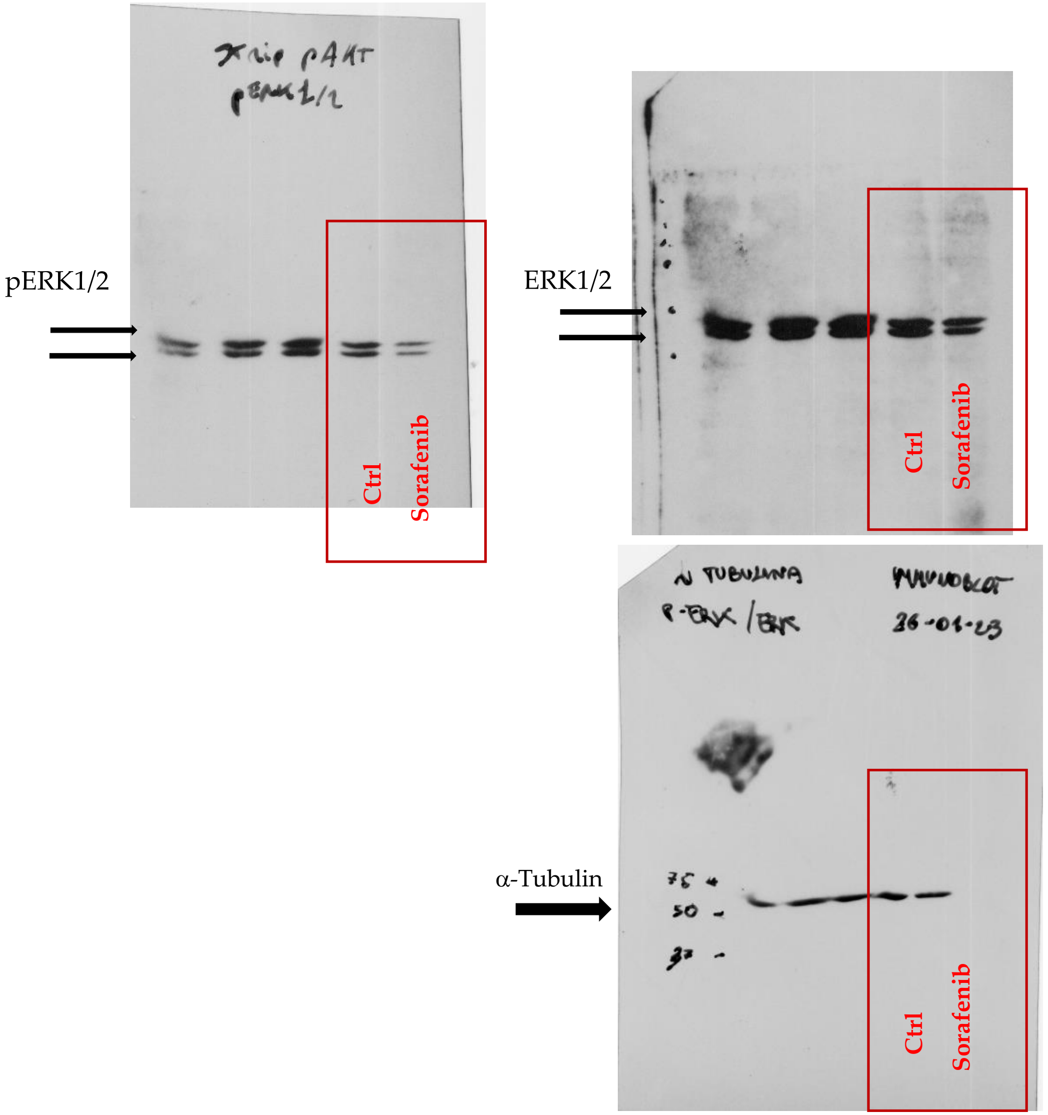

Figure 8 b

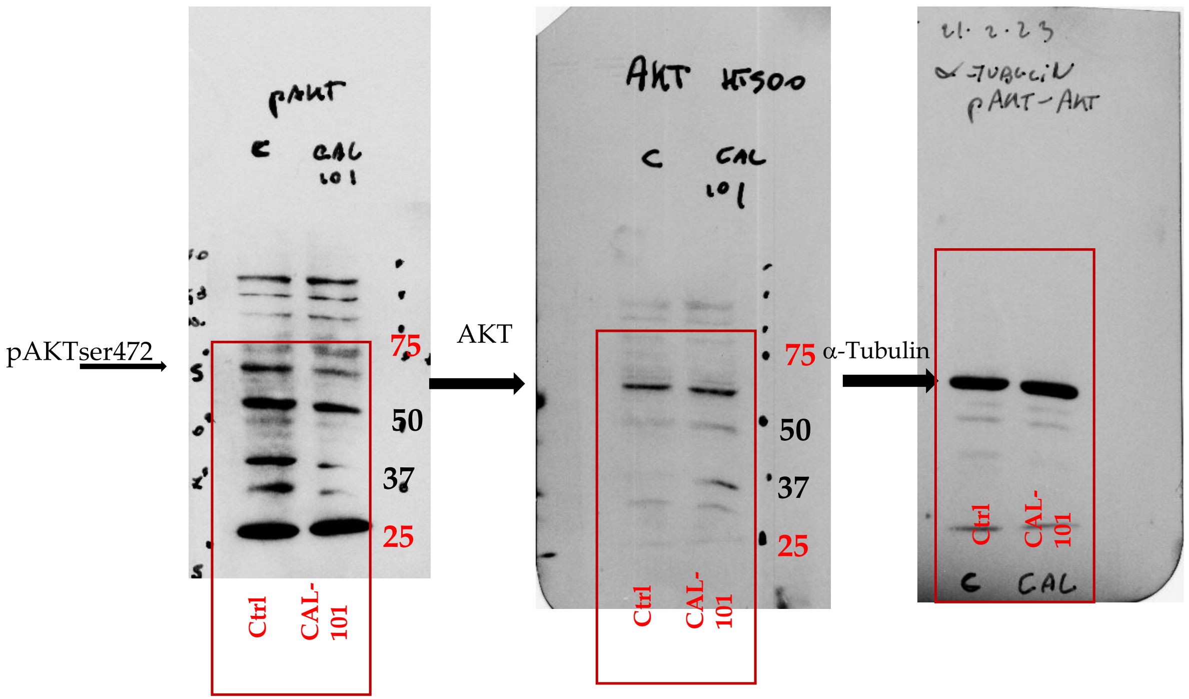

Figure S2

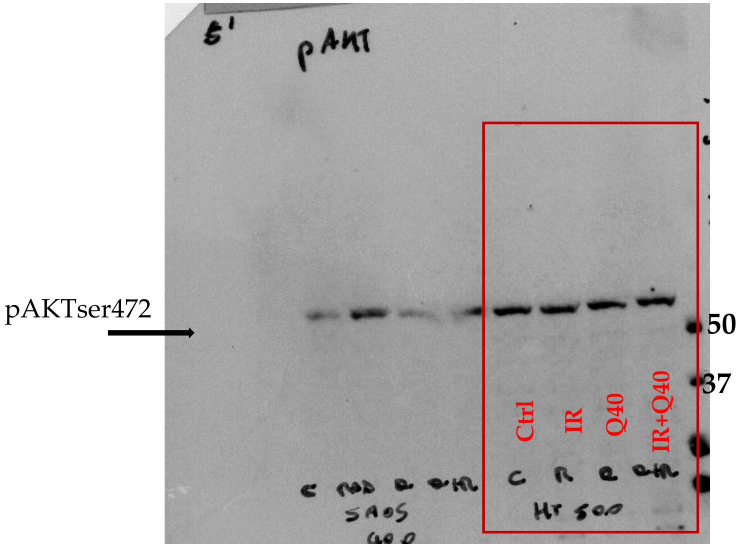
pAKTser472
50
37
Ctrl
IR
Q40
IR+Q40

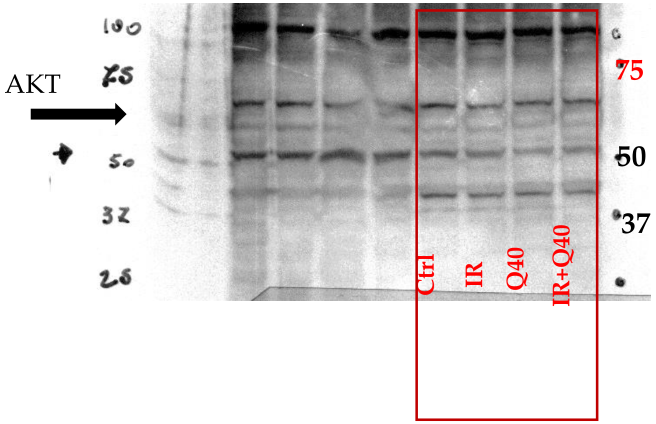
AKT
75
50
37
Ctrl
IR
Q40
IR+Q40

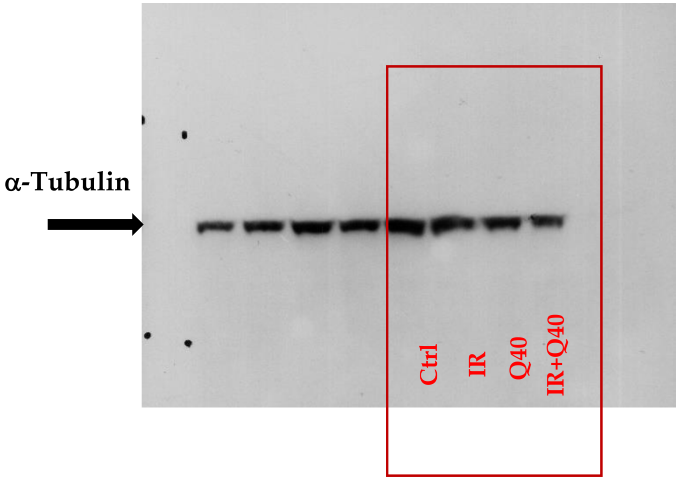
α-Tubulin
Ctrl
IR
Q40
IR+Q40
